# Unpacking flexible work and employee thriving: knowledge-motivation mediation and the boundary role of cognitive flexibility

**DOI:** 10.3389/fpsyg.2026.1701795

**Published:** 2026-04-22

**Authors:** Bingxin Xia, Mengze Zhang, Zhaoqi Li

**Affiliations:** 1 Department of Economics, Sejong University, Seoul, Republic of Korea; 2 Department of Business, Gachon University, Seongnam, Republic of Korea

**Keywords:** flexible work arrangements, thriving at work, knowledge acquisition, intrinsic motivation, cognitive flexibility

## Abstract

**Introduction:**

Remote collaboration and digital work have become increasingly prevalent, making flexible work arrangements (FWA) a critical strategy for enhancing organizational adaptability and employee effectiveness. However, the extent to which such institutional practices translate into positive employee outcomes depends on the activation of underlying psychological mechanisms.

**Methods:**

Drawing on Self-Determination Theory (SDT), this study develops a chained mediation model (institutional activation → knowledge acquisition → intrinsic motivation → thriving at work). Cognitive flexibility (CF) is incorporated as a dual-function adaptive mechanism. Data were collected from 427 knowledge workers in China using a three-wave time-lagged survey design.

**Results:**

The results indicate that FWA significantly promotes employees' thriving at work through sequential mediation of knowledge acquisition and intrinsic motivation. Furthermore, cognitive flexibility strengthens these transmission effects, serving as a critical moderator within the institution-cognition-motivation-behavior pathway.

**Discussion:**

This study extends SDT by enriching the relational pathway and identifying the cognitive boundary conditions of institutional influence. It also provides practical implications for organizational design, employee empowerment, and the development of adaptive capacity in digital work environments.

## 1 Introduction

The rapid advancement of artificial intelligence, big data, and collaborative technologies requires both organizations and employees to adapt and respond to structural transformations and environmental uncertainties at a faster pace (Li et al., 2025). As a result, traditional static task models are gradually being replaced by more flexible and adaptive work approaches that not only demonstrate resource agility and dynamic responsiveness but also better meet diverse work needs. In the context of normalized remote collaboration, flexible work arrangements (FWA)—as institutional practices that integrate temporal and spatial flexibility—have become a critical foundation for enhancing organizational adaptability and resilience, as well as promoting employee effectiveness and positive work behaviors.

Previous studies document that FWA not only helps improve organizational attractiveness, employee autonomy, and positive affect (Hornung et al., 2008; Onken-Menke et al., 2018; Timms et al., 2015) but is also accompanied by potential challenges such as increased cognitive load, blurred boundaries, and diluted social interactions (Kossek, 2016; Park et al., 2020; Pedersen and Lewis, 2012). These findings reveal a dual mechanism of facilitation and inhibition, thus highlighting the systemic ambivalence of flexible work structures (Wang and Xie, 2023).

Against this backdrop, thriving at work (TW), which is a composite psychological state in which employees experience both vitality and learning, has garnered increasing attention in both academic and managerial domains (Goh et al., 2022; Jiang et al., 2025; Spreitzer et al., 2005). Compared with traditional outcome indicators such as job satisfaction or performance, thriving reflects employees' ongoing developmental capacity and systemic adaptability within organizational contexts. TW not only embodies an individual's resource regeneration mechanism but also significantly contributes to enhancing an organization's overall innovativeness and operational resilience (Kleine et al., 2019; Miceli et al., 2021).

Empirical research has shown that TW can significantly enhance employees' proactivity, work engagement, and innovative behavior, while reducing tendencies toward burnout and turnover (Alikaj et al., 2021; Shahid et al., 2021; Xie et al., 2024). These positive changes, in turn, contribute to improving organizational efficiency and adaptability. Thus, fostering TW is not only a crucial bridge linking institutional design to organizational performance but also a key pathway for activating positive psychological resources and behavioral outcomes. Examining the mechanisms through which FWA influences TW is essential for understanding employees' psychological adaptation processes in flexible work contexts and for building more adaptive and resilient organizations.

This study is grounded in the core framework of Self-Determination Theory (SDT), which posits that the persistence and positivity of individual behavior stem from the fulfillment of three basic psychological needs: autonomy, competence, and relatedness (Deci and Ryan, 1985; Ryan and Deci, 2000). From this perspective, FWA is conceptualized as systemic resource activation mechanisms through which employees' internal psychological processes are stimulated and ultimately translated into positive behavioral outcomes. Specifically, FWA provide employees with greater autonomy over their tasks, ultimately allowing them to independently decide when, where, and how to work, and thereby fulfilling their need for autonomy (Liu et al., 2024). At the same time, the abundant information resources and learning opportunities embedded within flexible work systems facilitate knowledge acquisition (KA) and skill enhancement, thus reinforcing employees' sense of competence (Jiang et al., 2023). The fulfillment of these two psychological needs jointly promotes intrinsic motivation (IM), which in turn fosters a higher level of TW.

Building upon the satisfaction of basic psychological needs, this study introduces KA and IM as key mediating variables to further reveal the psychological mechanism through which FWA promotes thriving. First, FWA enhances employees' exposure to novel knowledge and diverse information, thereby strengthening their task control and cognitive activation. The continuous inflow of knowledge contributes to employees' perceived growth and meaningfulness, which ignites stronger IM. Motivated by this internal drive, employees exhibit greater vitality and a stronger willingness to engage in continuous learning. Ultimately, this chained psychological mechanism, which is comprised of KA and IM, enables employees to achieve higher levels of thriving at work under flexible work conditions.

Within this systemic relational chain, employees' cognitive flexibility (CF) emerges as a critical moderating factor that determines whether institutional resources can be absorbed and transformed effectively. As a cognitive capacity that reflects individuals' information processing adaptability and coping strategies (Cañas et al., 2003), CF not only shapes employees' interpretations of complex institutional inputs but also functions as a psychological mechanism that complements the fulfillment of the relatedness need within the SDT framework, thereby compensating for relational gaps often induced by flexible work arrangements.

Specifically, employees with high CF are more capable of adjusting their cognitive and communication strategies in response to diverse work environments and complex social interactions (Kwon and Kim, 2025). Such employees are better equipped to identify and leverage both institutional and interpersonal resources, which enhances the quality of social interactions and fosters a sense of belonging. This, in turn, helps fulfill the need for relatedness and further strengthens the motivational effects of their intrinsic drive. By contrast, employees with low CF are more prone to cognitive rigidity and social detachment, which may hinder the motivational and behavioral benefits derived from institutional support, thereby weakening the positive impact of FWA on IM and thriving at work.

In summary, this study aims to reveal how FWA translate into employees' developmental outcomes through psychological mechanisms by constructing a chained mediation model of “institutional activation → knowledge acquisition → intrinsic motivation → thriving at work.” CF is incorporated as a moderating variable to examine its boundary role in the institutional influence pathway, thereby highlighting the importance of individual differences in the transformation of institutional resources.

To ensure the contextual relevance and external validity of the proposed model, we conducted a three-wave survey among 427 knowledge workers with flexible work experience. The sample covered cognitively intensive sectors, including information and communications technology (ICT), advanced manufacturing, internet services, and research and education. These industries not only demand high levels of cognitive ability but are also highly sensitive to institutional changes, thus providing an appropriate context and robust empirical basis for testing the proposed psychological and behavioral transformation mechanisms.

This study makes three key contributions. First, it responds to the theoretical call for integrating multilevel resources and behavioral mechanisms by proposing a chained mediation model that follows the logic of “input-absorption-transformation-output,” thereby enriching the psychological explanatory framework of FWA. Second, beyond autonomy and competence, this study incorporates the often-overlooked need for relatedness in flexible work contexts. We conceptualize CF as a dual-function adaptation mechanism: on the one hand, it enhances employees' capacity to interpret and utilize complex institutional resources; on the other hand, it compensates for the relational erosion caused by remote and flexible work, thereby partially fulfilling the need for relatedness within the SDT framework. Through this perspective, the study highlights the boundary role of individual cognitive traits in the transmission of institutional influences. Third, by focusing on knowledge workers as a distinct occupational group, this study explores their motivational processes and psychological need fulfillment under FWA, filling a research gap on the heterogeneity of knowledge workers and their resource transformation patterns, and offering practical guidance for designing more targeted institutional support and motivational strategies for this group.

## 2 Theoretical background and hypotheses

### 2.1 Self-determination theory

SDT posits that the fulfillment of an individual's basic psychological needs is a fundamental prerequisite for sustained positive behavior and personal growth (Ryan and Deci, 2000). Unlike traditional forms of external motivation, IM arises from the satisfaction of three core needs of autonomy, competence, and relatedness, thus making it more stable and enduring over time (Deci and Ryan, 2008; Van den Broeck et al., 2016). Within the context of FWA, employees are granted greater autonomy in choosing how they work and are exposed to diverse and abundant knowledge resources. These conditions facilitate the satisfaction of autonomy and competence needs, thereby enhancing knowledge absorption and motivational transformation processes, which ultimately promote higher levels of TW (Jiang et al., 2023; Porath et al., 2022).

Grounded in SDT, this study explores how FWA promotes employee thriving through the sequential pathway of psychological need satisfaction and IM. Furthermore, our model incorporates CF as an indirect mechanism for fulfilling the need for relatedness, thereby extending the theoretical applicability and explanatory power of SDT in the context of flexible work systems.

### 2.2 Flexible work arrangements and employee thriving

FWA enhances employee thriving, particularly among knowledge workers, by offering flexibility along both temporal and spatial dimensions. From a temporal perspective, FWA allows employees to schedule their working hours based on personal life rhythms and task requirements (Hayman, 2009). This temporal autonomy significantly strengthens employees' sense of control and psychological freedom at work (Wu et al., 2024), ultimately laying a critical psychological foundation for activating vitality and continuous learning. When employees are empowered to determine when they work, they are more likely to apply their professional competencies to actual tasks flexibly, thereby experiencing heightened engagement and psychological satisfaction (Fuller and Unwin, 2010; Morgeson et al., 2005).

From a spatial perspective, FWA breaks the physical constraints of traditional office environments through mechanisms such as remote work and working from home. This enables knowledge workers to select the most suitable work setting according to their cognitive preferences and task characteristics (Gerdenitsch et al., 2015). Such spatial flexibility effectively reduces the stress caused by commuting or rigid office settings while enhancing employees' discretionary control over their tasks and their ability to adapt to diverse environments (Kossek et al., 2015). Collectively, these conditions foster greater work vitality, self-directed learning, and a more positive overall work experience (Radovan, 2024; Zhou et al., 2024). Accordingly, this study formulates the following hypothesis:

H1: flexible work arrangements have a positive effect on employee thriving at work.

### 2.3 Mediating role of knowledge acquisition

KA refers to the process by which employees actively identify, absorb, and integrate task-relevant information and knowledge resources during their work, through mechanisms such as institutional empowerment, autonomous exploration, and social interaction. This process involves not only the intake of external knowledge, but also emphasizes active processing, contextual adaptation, and internalization of knowledge at the individual level (Argote et al., 2003; López et al., 2006). Unlike passive information reception, KA reflects the dynamic operation of employees' cognitive systems and serves as a key mechanism for continuous development and proactive capability building (Barba-Aragón and Jiménez-Jiménez, 2024; Huber, 1991; Nonaka and Toyama, 2003).

Within this framework, an institutional environment characterized by openness and autonomy becomes a critical external enabler of KA. By breaking the temporal and spatial constraints of traditional work, FWA provides knowledge workers with greater task control and environmental adaptability, thereby creating a favorable institutional foundation for KA. Such highly flexible structures not only enhance employees' control over task pace and content, but also significantly expand their opportunities to access new knowledge, engage in diverse work scenarios, and participate in cross-domain learning (Jiang et al., 2023).

Furthermore, KA is not merely a process of processing external information, rather, KA is also a continuous renewal of individual cognitive structures and an ongoing optimization of capability systems. This cognitive transformation mechanism is directly reflected in employees' TW. As knowledge accumulates and skills are systematically integrated, employees develop stronger problem-solving abilities, professional judgment, and responsiveness to dynamic tasks, thereby enhancing their adaptive capacity and cognitive agility (Baroody, 2013). This sense of progress and mastery contributes to a deeper psychological connection to work and a stronger career identity, which in turn promotes sustained engagement and a positive psychological state (Zhu and Song, 2022).

In summary, KA serves as a pivotal mediating mechanism between the institutional structure and individual cognitive systems. Furthermore, KA bridges organizational support and employees' developmental experience, consequently playing both a foundational and facilitating role in the process of achieving TW. Accordingly, this study formulates the following hypothesis:

H2: knowledge acquisition mediates the relationship between flexible work arrangements and employee thriving at work. Specifically, FWA enhance employees' access to knowledge, strengthen their cognitive resources and professional capabilities, and thereby promote higher levels of thriving.

### 2.4 Mediating role of intrinsic motivation

IM refers to an individual's psychological inclination to voluntarily engage in activities out of interest, curiosity, or inherent satisfaction derived from the task itself, rather than being driven by external rewards or pressure. Such motivation represents an internalized valuation of the activity and is characterized as a self-directed and sustainable form of motivation (Deci and Ryan, 2008).

According to SDT, FWA is considered a vital institutional mechanism for fostering IM among employees. By offering enhanced temporal flexibility and spatial autonomy, FWA significantly improves employees' sense of control, self-worth, and responsibility over their tasks, while also strengthening their identification with and acceptance of organizational systems (Azeem and Kotey, 2023; Peretz et al., 2018). This structurally enabled experience of autonomy leads employees to perceive their work as a platform for self-development and capability building rather than merely a site for performing externally imposed duties (Weideman and Hofmeyr, 2020). Moreover, such “voluntary engagement” satisfies basic psychological needs and provides a psychological and institutional foundation for the activation of IM (Gagné et al., 2015).

More importantly, a growing body of research has identified IM as a key psychological driver of employee TW. TW refers to a positive psychological state in which employees experience both vitality and learning in their work processes. High levels of IM promote proactive exploration, continuous growth, and meaningful contribution in daily tasks. These behaviors contribute to the formation of a self-reinforcing psychological—behavioral cycle, which enables employees to sustain a positive and engaged work state over time (Han et al., 2024; Kleine et al., 2019; Liu et al., 2021).

In summary, FWA does not directly enhance employee thriving through behavioral enforcement; rather, FWA stimulates IM by fulfilling employees' basic psychological needs, which in turn fosters continuous engagement, self-improvement, and personal growth, ultimately leading to higher levels of TW. Accordingly, this study formulates the following hypothesis:

H3: intrinsic motivation mediates the relationship between flexible work arrangements and employee thriving. Specifically, FWA enhances employees' intrinsic motivation, which in turn promotes greater thriving at work.

### 2.5 Chain mediating role of knowledge acquisition and intrinsic motivation

FWA enhances employees' sense of autonomy by offering greater flexibility in both time and space, thereby allowing knowledge workers to exercise increased discretion over task scheduling, work pace, and execution methods. By breaking the constraints of traditional rigid work structures, FWA empowers employees to plan their activities in alignment with their interests and goals, thereby stimulating personal initiative and exploratory intentions (Wang et al., 2021).

In such highly autonomous work environments, employees are more inclined to proactively acquire new knowledge, expand their skill boundaries, and deepen their understanding of task complexity and contextual dynamics. As a critical cognitive mediator linking institutional features with psychological resource perceptions, a stable process of KA not only broadens employees' cognitive scope and professional judgment, but also strengthens their sense of competence and self-efficacy (Lambert et al., 2008).

SDT posits that when employees' basic psychological needs for autonomy and competence are fulfilled, their IM is significantly enhanced (Deci and Ryan, 2008). This heightened IM drives individuals to engage in continuous learning and growth with greater enthusiasm and proactivity. Consequently, employees experience stronger vitality and learning orientation, which are the dual components of TW, which is a positive psychological state reflecting both energy and developmental experience (Iqbal et al., 2024; Spreitzer and Hwang, 2019).

In summary, FWA enhances employees' perceived autonomy in work design and strengthens their sense of competence through enriched KA, which in turn fuels IM and ultimately leads to higher levels of TW. Based on this logic, we propose a chain mediation model that begins with institutional characteristics, flows through cognitive evaluation and motivational activation, and culminates in employees' positive work states, thus forming a systemic “institution—cognition—motivation—behavior” pathway. Accordingly, this study formulates the following hypothesis:

H4: knowledge acquisition and intrinsic motivation jointly mediate the relationship between flexible work arrangements and employee thriving at work, thus forming a sequential (chain) mediation pathway.

### 2.6 Moderating role of cognitive flexibility

FWA provides knowledge workers with greater temporal and spatial freedom, thereby facilitating access to diverse knowledge sources and enhancing information flow. However, the extent to which employees can effectively identify and utilize these institutional resource opportunities largely depends on individual cognitive traits, particularly the level of CF.

CF refers to an individual's capacity to adjust cognitive strategies, accommodate diverse information structures, and rapidly switch mental frameworks when faced with complex, dynamic, or uncertain environments (Martin and Rubin, 1995). In flexible work settings, formal knowledge transfer mechanisms are often weakened, while informal networks become critical channels for KA (Becker, 2007; Karhapää et al., 2025; Taskin and Bridoux, 2010). Under such conditions, employees with high CF are more adept at detecting latent information opportunities and actively seeking and integrating external knowledge resources. Specifically, cognitively flexible employees are better equipped to adapt to diverse social contexts, such as remote collaboration and cross-departmental communication, and to proactively build interactive relationships with different stakeholders. This enables them to expand their knowledge access radius and improve their KA efficiency (Kwon and Kim, 2025; Ouellette et al., 2024).

Conversely, employees with low CF may struggle to adjust to evolving work environments and fail to identify developmental opportunities or build effective connections. Consequently, they may underutilize institutional resources, potentially leading to negative outcomes such as burnout and decreased performance (Kraimer et al., 2022).

Therefore, CF not only enhances employees' ability to process and integrate information under flexible institutional conditions but also strengthens their capacity to construct and utilize social relationship resources. Thus, CF serves as a critical moderating variable that links institutional resources with knowledge-related behaviors. Accordingly, this study proposes the following hypothesis:

H5: cognitive flexibility positively moderates the relationship between flexible work arrangements and knowledge acquisition, such that the positive effect of FWA on knowledge acquisition is stronger when employees exhibit higher levels of cognitive flexibility.

### 2.7 Moderating role of cognitive flexibility in the relationship between FWA and intrinsic motivation

As a work system that emphasizes temporal and spatial autonomy, FWA enhances employees' discretion over task scheduling, pace management, and work methods. This structural autonomy provides an institutional foundation for stimulating employees' personal initiative and psychological engagement. However, the freedom and flexibility released by the institutional environment do not automatically translate into positive psychological responses. A key determinant of this process is whether employees possess sufficient cognitive capacity to interpret, utilize, and internalize the institutional resources provided by FWA, thus enabling the activation of IM.

Defined as the capacity to process complex information, adjust thinking strategies, and adapt to environmental changes (Martin and Rubin, 1995), CF plays a crucial moderating role in the psychological effects of flexible systems. Employees with high CF are more likely to interpret the empowering intentions behind FWA amidst uncertainty and self-management demands. They are also more capable of setting personal goals, optimizing task structures, and generating self-driven expectations (Kubicek et al., 2022, 2021; Kwon and Kim, 2025). This high level of cognitive adaptability enables them to transform institutional freedom into experiences of autonomy and enhanced competence, thereby effectively stimulating IM (Deci and Ryan, 2008).

By contrast, employees with low CF may struggle with the ambiguity and self-regulatory demands of flexible work systems. They may experience a sense of “structural vacuum,” find forming clear behavioral goals or feelings of autonomy difficult, and as a result, the psychological motivational potential of institutional resources may be significantly weakened (Koenders and Lostakova, 2025).

Therefore, as a key individual difference variable, CF determines whether flexible work arrangements can be effectively translated into psychological satisfaction and IM. CF serves as a crucial moderating factor linking institutional design with employee psychological agency. Based on this rationale, the following hypothesis is proposed:

H6: Cognitive flexibility positively moderates the relationship between flexible work arrangements and intrinsic motivation, such that the positive effect of FWA on intrinsic motivation is stronger when employees exhibit higher levels of cognitive flexibility.

### 2.8 Moderated mediation role of cognitive flexibility

Grounded in SDT, this study constructs a chained mediation model—“Flexible Work Arrangements (FWA) → Knowledge Acquisition → Intrinsic Motivation → Thriving at Work”—to systematically reveal how organizational systems foster employees' sustained developmental states by activating core psychological mechanisms. According to SDT, autonomy, competence, and relatedness are the three essential psychological needs that drive the emergence of IM (Deci and Ryan, 2008).

Within this framework, FWA enhances employee autonomy by offering greater temporal and spatial control, thereby strengthening their discretion over task execution and pace. Simultaneously, the provision of learning opportunities and access to knowledge resources enhances employees' professional capabilities and task mastery, effectively fulfilling their need for competence. Due to physical separation, reduced face-to-face interaction, and increasingly loose team structures, employees have fewer opportunities to obtain informal social cues and affective feedback. This, in turn, may weaken interpersonal trust and social support mechanisms, thereby making the fulfillment of relatedness needs more uncertain and challenging (Franken et al., 2021; Golden et al., 2008; Cooper and Kurland, 2002). In this context, employees' ability to proactively build interpersonal connections, integrate into informal communication networks, and obtain social support becomes critical for the successful psychological internalization of institutional incentives.

Against this backdrop, CF, as a psychological trait reflecting individuals' adaptive capacity and cognitive strategy adjustment in response to environmental changes, may serve as a substitute mechanism for fulfilling the need for relatedness. Previous research demonstrates that individuals' cognitive traits significantly influence their learning processes, motivation formation, and behavioral responses (Luria et al., 2021). Employees with high CF demonstrate greater sensitivity and adaptability in complex social situations. Furthermore, they are able to swiftly identify informal cues, flexibly adjust their communication and collaboration strategies (Cañas et al., 2003), and proactively build cross-boundary social networks that enhance interpersonal connections and interactive engagement (Radovan, 2024; Roy and Dugal, 1998; Ryan and Deci, 2000). This cognitively driven social construction ability enhances employees' efficiency in terms of identifying and absorbing institutional resources and indirectly facilitates the fulfillment of relatedness in the absence of traditional emotional support.

From the perspective of SDT, although relatedness is traditionally achieved through affective bonding and belongingness, its realization under highly autonomous and low-structure systems is increasingly dependent on a hybrid mechanism of cognitive adaptation and social construction. As such, CF serves as a cognitive pathway for satisfying relatedness, enhancing the transmission efficiency from institutional inputs to knowledge behaviors and motivational outcomes, and ultimately influencing employees' developmental states.

In summary, CF not only moderates the direct effects of FWA on KA and IM but also functions as a boundary condition for the entire “institution—psychology—behavior” mechanism. CF enhances the stability and depth of transformation within the chain mediation pathway. Therefore, we propose the following hypothesis:

H7: Cognitive flexibility moderates the chained mediation effect of knowledge acquisition and intrinsic motivation in the relationship between flexible work arrangements and employee thriving. Specifically, when CF is high, employees are more likely to achieve substitute fulfillment of relatedness, thereby strengthening the indirect effects of FWA on thriving through knowledge acquisition and intrinsic motivation.

**Figure 1:**
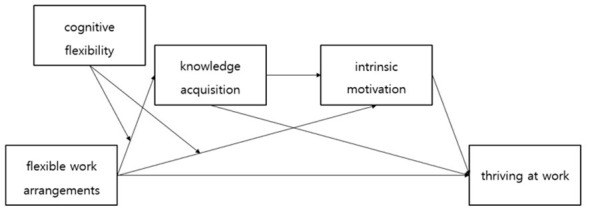
Research model.

Based on these hypotheses, we developed the following research model (Figure 1).

## 3 Method

### 3.1 Data collection

#### 3.1.1 Target population and sampling framework

This study targeted knowledge workers in Southern China who had prior experience with FWA. The sample was drawn primarily from sectors such as ICT, high-tech creative industries, Internet enterprises, and research and education institutions. These industries were selected because of their relatively advanced adoption of FWA and the generally high cognitive complexity of their work tasks. To enhance the representativeness and heterogeneity of the sample, we implemented a stratified sampling design across five dimensions: gender, age, education level, job position, and tenure. This approach ensured a multilevel sample structure and broad coverage of diverse organizational backgrounds and employee characteristics, thereby capturing the psychological effects of FWA across varied individual profiles.

#### 3.1.2 Data collection procedure

To reduce common method bias, a three-wave time-lagged survey design was employed, with each wave spaced approximately 2 weeks apart. Data were collected between October and November 2024 via Credamo, which is a professional online survey platform widely used in academic research in China. Credamo provides functionality comparable to Amazon Mechanical Turk, including structured sampling, participant tracking, and data quality control.

This study was conducted in full compliance with the Exempt Research Ethics Guidelines established by the Institutional Review Board (IRB). Prior to data collection, the research team provided all participants with a detailed explanation of the study, clearly informing them that the survey would be administered in three phases, that participation was entirely voluntary, and that they could withdraw at any time without penalty. All responses were collected anonymously and treated with strict confidentiality. Informed consent was obtained from all participants without any external pressure.

In the first wave (T1), 600 knowledge workers were invited to complete a survey measuring their perceptions of FWA, CF, and demographic information. In the second wave (T2), 516 valid responses were collected, in which participants reported their levels of KA and IM. In the third wave (T3), respondents who had completed the previous two waves filled out scales related to TW.

After matching the survey codes across the three waves and screening for data quality (e.g., removing incomplete, inconsistent, or overly rapid responses), a final sample of 427 valid cases was retained, which resulted in an overall effective response rate of 59.7%.

#### 3.1.3 Descriptive statistics

This study conducted statistical analyses of respondents' demographic characteristics, such as gender and age, using SPSS 27.0. A total of 427 valid responses were collected. In terms of gender, males constituted the majority, accounting for 70.3% of the sample. The age distribution was concentrated in the 30–39 (38.9%) and 20–29 (32.8%) age brackets, reflecting a predominance of young and middle-aged participants. Regarding education, 81.9% held a bachelor's degree or above, indicating a high level of educational attainment. For work experience, more than half of the respondents (54.1%) had been employed for 6–15 years, demonstrating substantial professional experience. In terms of job position, the majority were frontline employees (56.7%) or first-line supervisors (34.0%), primarily engaged in professional roles and entry-level management. Overall, the sample possessed strong professional knowledge and work experience, making it highly representative of knowledge workers.

### 3.2 Measurement instruments

This study employed several well-established measurement scales. All original items were adapted from English-language sources, and the translation process followed Brislin's (1970) recommended back-translation procedure to ensure content equivalence between the Chinese and English versions. Unless otherwise noted, all variables were measured using a five-point Likert scale (1 = strongly disagree, 5 = strongly agree). Composite scores were computed as the mean of all corresponding items. The specific measures are as follows.

#### 3.2.1 Flexible work arrangements

FWA was measured using a four-item scale developed by Hornung et al. (2008) and adapted to the Chinese context by Liu and Wang (2022). The scale assesses employees' perceived flexibility in both time and space dimensions. Sample items include “The company's employees can flexibly arrange their working hours according to their actual situation.” and “Employees can work from home or remotely.”

#### 3.2.2 Cognitive flexibility

CF was assessed using a 12-item scale developed by Martin and Rubin (1995), which evaluates an individual's capacity to shift thinking strategies and adapt to complex environments. The scale includes reverse-coded items. Sample items include “I am open to new and unusual situations.” and “I have many possible ways of behaving in any given situation.”

#### 3.2.3 Knowledge acquisition

KA was measured using the four-item scale proposed by Pérez López et al. (2005), which captures the extent to which employees acquire professional knowledge and information from external sources. Sample items include “I maintain close contact and communication with external technical experts to gain professional knowledge.” and “I actively participate in formal or informal groups composed of external individuals to obtain job-related knowledge.”

#### 3.2.4 Intrinsic motivation

IM was measured using a six-item scale developed by Kuvaas et al. (2017), which reflects employees' internal drive to engage in work for its own sake. Sample items include “My work is so interesting that it serves as its own motivation.” and “My job often provides me with feelings of excitement and challenge.”

#### 3.2.5 Thriving at work

TW was assessed using the 10-item scale developed by Porath et al. (2012), which comprises two dimensions: learning and vitality. Sample items include: Learning dimension: “I often find myself learning new things at work.” Vitality dimension: “I look forward to each workday as a new opportunity and challenge.”

#### 3.2.6 Control variables

To account for potential individual differences, we included the following control variables: gender, age, educational level, tenure, and job position. These variables were collected through a self-report method and incorporated into the model as categorical variables to control for their possible influence on employees' psychological states and behavioral outcomes.

## 4 Results

### 4.1 Reliability analysis

Cronbach's alpha coefficients were calculated to ensure the internal consistency of the measurement scales. The reliability coefficients for all core variables exceeded the threshold of 0.80, thereby demonstrating a high level of reliability across the constructs. Specifically, the Cronbach's α values were as follows: Flexible Work Arrangements (α = 0.874), Knowledge Acquisition (α = 0.897), Intrinsic Motivation (α = 0.909), Cognitive Flexibility (α = 0.938), and Thriving at Work (α = 0.915), all indicating excellent internal consistency.

Moreover, the two subdimensions of thriving, Learning (α = 0.883), and Vitality (α = 0.881), also displayed high reliability. These findings suggest that the scales used in this study possess strong measurement stability and reliability.

### 4.2 Validity analysis

To assess the construct validity of the measurement model, confirmatory factor analysis (CFA) was conducted using AMOS 23.0. The results revealed a good fit for the hypothesized five-factor model, with the following indices:


$$\begin{array}{l} \chi^{\text{2}}/\text{df}=1.382<3,\text{SRMR}=0.035<0.05,\text{TLI}=0.975,\\ \text{CFI}=0.977,\text{RMSEA}=0.030<0.05. \end{array}$$

These values indicate that the measurement model has excellent structural validity.

When compared with alternative models, including four-, three-, two-, and one-factor models, the proposed five-factor model exhibited significantly better fit, thereby further supporting the appropriateness of the construct structure (see Table 1).

**Table 1:** Comparison of measurement models.

Model	χ^2^	df	χ^2^/df	SRMR	TLI	CFI	RMSEA
Five factor model	804.033	582	1.382	0.035	0.975	0.977	0.030
Four factor model	1558.325	586	2.659	0.059	0.890	0.898	0.062
Three factor model	1949.352	589	3.310	0.062	0.847	0.857	0.074
Two factor model	4455.458	591	7.539	0.205	0.568	0.595	0.124
Single factor model	5607.393	594	9.440	0.172	0.442	0.474	0.141

Five-factor model: FWA, KA, IM, CF, TW; Four-factor model: FWA, KA+IM, CF, TW; Three-factor model: FWA+KA+IM, CF, TW; Two-factor model: FWA+KA+IM+CF, TW; and Single-factor model: FWA+KA+IM+CF+TW.

### 4.3 Common method bias and multicollinearity diagnostics

Harman's single-factor test was used to assess the potential for common method bias. The first unrotated principal component accounted for 32.088% of the total variance, which is well below the commonly accepted threshold of 40%. This indicates that common method bias is not a serious concern in this study.

Additionally, to diagnose potential multicollinearity, variance inflation factors (VIF) were examined for all key independent variables. The results indicated that VIF values ranged from 1.076 to 1.749, well below the conservative threshold of 10, suggesting that multicollinearity was not a serious concern in the present study.

### 4.4 Correlation analysis and discriminant validity

Pearson correlation analysis revealed significant positive correlations among the key variables, excluding some demographic control variables. These findings support the assumptions required for subsequent regression analyses. In particular, Flexible Work Arrangements displayed moderately strong correlations with Knowledge Acquisition (*r* = 0.548, *p* < 0.01), Intrinsic Motivation (*r* = 0.540, *p* < 0.01), and Thriving at Work (*r* = 0.567, *p* < 0.01), thus providing preliminary evidence for the theoretical linkages among the core constructs.

In addition, the square roots of the average variance extracted (AVE) for each latent variable (displayed on the diagonal of the correlation matrix) were all greater than the corresponding inter-construct correlation coefficients, which indicates acceptable discriminant validity of the measurement model.

Taken together, the results of the correlation analysis provide a solid empirical basis for subsequent regression analyses and mediation effect testing (see Table 2).

**Table 2:** Correlation matrix and discriminant validity.

Variables	(1)	(2)	(3)	(4)	(5)	(6)	(7)	(8)	(9)	(10)
(1) Gender	1									
(2) Age	−0.031	1								
(3) Education	−0.072	−0.024	1							
(4) Tenure	0.004	0.751^**^	−0.158^**^	1						
(5) Position	−0.014	0.389^**^	0.005	0.450^**^	1					
(6) FWA	0.032	0.039	−0.065	0.056	−0.007	(0.797)				
(7) KA	0.008	−0.024	0.036	−0.006	0.031	0.548^**^	(0.828)			
(8) IM	0.076	0.024	−0.003	0.054	0.018	0.540^**^	0.463^**^	(0.791)		
(9) CF	0.046	−0.048	−0.114^*^	0.007	−0.083	0.264^**^	0.168^**^	0.130^**^	(0.749)	
(10) TW	0.080	0.045	−0.056	0.095^*^	−0.011	0.567^**^	0.516^**^	0.577^**^	0.220^**^	(0.775)

^*^p < 0.05, ^**^p < 0.01, ^***^p < 0.001; FWA, flexible work arrangement; KA, knowledge acquisition; IM, intrinsic motivation; CF, cognitive flexibility; and TW, thriving at work.

### 4.5 . Hypothesis testing

Hierarchical regression analysis was conducted to test the proposed hypotheses. As shown in Model 3, Flexible Work Arrangements had a significant positive effect on Thriving at Work (β = 0.503, *p* < 0.001), thus providing strong support for H1 (see Table 3).

**Table 3:** Results of hierarchical regression analysis.

Variables	KA	IM	TW	TW
	Model 1	Model 2	Model 3	Model 4
Constant	0.916^*^	0.992^**^	1.412^***^	0.899^**^
Gender	−0.015	0.146	0.124	0.085
Age	−0.089	−0.033	−0.055	−0.024
Education	0.117	0.043	0.003	−0.038
Tenure	−0.007	0.050	0.093	0.080
Position	0.105	−0.002	−0.055	−0.080
FWA	0.620^***^	0.407^***^	0.503^***^	0.233^***^
KA		0.213^***^		0.182^***^
IM				0.292^***^
*R* ^2^	0.311	0.337	0.332	0.465
Adjust *R*^2^	0.301	0.326	0.322	0.455
*F*	31.533	30.381	34.747	45.454
*p*	0.000	0.000		

^*^p < 0.05, ^**^p < 0.01, ^**^p < 0.001; FWA, flexible work arrangement; KA, knowledge acquisition; IM, intrinsic motivation; and TW, thriving at work.

A bootstrapping procedure with 5,000 resamples was used to test the significance of the mediation pathways. The total, direct, and total indirect effect of Flexible Work Arrangements (FWA) on Thriving at Work was 0.503, 0.233, and 0.270, respectively, with all 95% confidence intervals (CIs) excluding zero, thus indicating significant mediation effects.

Furthermore, as shown in Table 4, all three mediation paths are statistically significant. Specifically: FWA indirectly influenced thriving through Knowledge Acquisition [indirect effect = 0.113, 95% CI = (0.062, 0.165)]. FWA also had an indirect effect on thriving through Intrinsic Motivation [indirect effect = 0.119, 95% CI = (0.073, 0.174)]. Additionally, FWA influenced thriving via a sequential mediation path through Knowledge Acquisition → Intrinsic Motivation [indirect effect = 0.039, 95% CI = (0.018, 0.063)]. Since none of the CIs contained zero, all three mediation effects were statistically significant, thus providing strong support for H2, H3, and H4.

**Table 4:** Bootstrap analysis of mediation effects.

Pathway	Effect	SE	95% CI lower	95% CI upper	Total effect
Total effect	0.503	0.036	0.432	0.574	
Direct effect	0.233	0.042	0.150	0.315	46.30%
Total indirect effect	0.270	0.033	0.207	0.335	53.72%
FWA → KA → TW	0.113	0.026	0.062	0.165	22.41%
FWA → IM → TW	0.119	0.025	0.073	0.174	23.62%
FWA → KA → IM → TW	0.039	0.012	0.018	0.063	7.67%

FWA, flexible work arrangement; KA, knowledge acquisition; IM, intrinsic motivation; and TW, thriving at work.

#### 4.5.1 Test of moderating effects

Prior to conducting the moderation analysis, all predictor variables involved in the construction of interaction terms were mean-centered to reduce potential multicollinearity (Aiken, 1991). In addition, the moderating effects were tested using hierarchical regression analyses, in which control variables, main effects, and interaction terms were entered sequentially.

Moderating Effect of Cognitive Flexibility on the Relationship Between Flexible Work Arrangements and Knowledge Acquisition.

We tested the moderating effect of Cognitive Flexibility on the relationship between Flexible Work Arrangements (FWA) and Knowledge Acquisition. The interaction term FWA × Cognitive Flexibility significantly and positively predicted knowledge acquisition (β = 0.437, *p* < 0.001), thereby providing support for H5.

**Figure 2:**
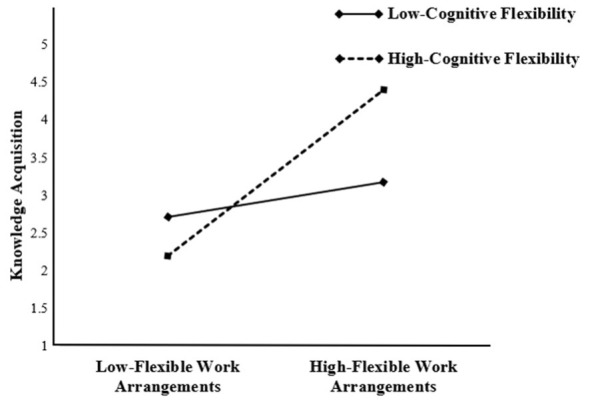
Interaction effect of cognitive flexibility on FWA and KA.

Furthermore, the simple slope analysis presented in Table 5 and Figure 2 indicates that the positive effect of FWA on KA becomes significantly stronger as the level of CF increases.

**Table 5:** Moderating effect of cognitive flexibility.

Level	Regression coefficient	SE	*t*	*p*	95% CI lower	95% CI upper
Low	0.311	0.055	5.664	0.000	0.203	0.420
Mean	0.666	0.044	15.240	0.000	0.580	0.751
High	1.020	0.064	16.058	0.000	0.895	1.145

Moderating Effect of Cognitive Flexibility on the Relationship Between Flexible Work Arrangements and Intrinsic Motivation.

We also examined the moderating effect of Cognitive Flexibility on the relationship between FWA and Intrinsic Motivation. The interaction term FWA × Cognitive Flexibility had a significant positive effect on IM (β = 0.324, *p* < 0.001), thus supporting H6.

**Figure 3:**
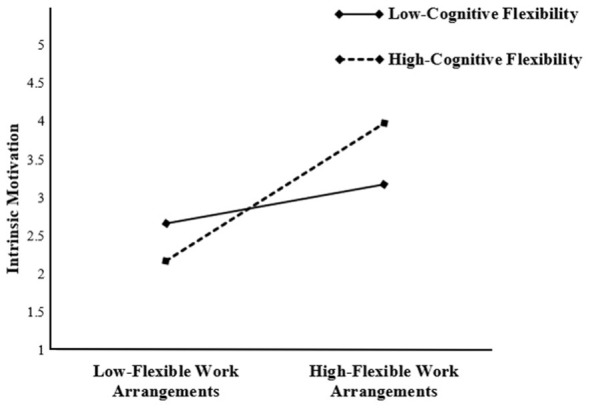
Interaction effect of cognitive flexibility on FWA and IM.

In addition, the simple slope analysis in Table 6 and Figure 3 further indicates that the positive impact of FWA on IM becomes significantly stronger at higher levels of CF.

**Table 6:** Moderating effect of cognitive flexibility.

Level	Regression coefficient	SE	*t*	*p*	95% CI lower	95% CI upper
Low	0.320	0.051	6.249	0.000	0.220	0.421
Mean	0.583	0.041	14.315	0.000	0.503	0.663
High	0.845	0.059	14.280	0.000	0.729	0.962

#### 4.5.2 Test of the moderated chain mediation effect

To examine whether Cognitive Flexibility moderated the chained mediation effect, a bootstrapping procedure with 5,000 resamples was conducted using a 95% bias-corrected CI. The results are presented in Table 7.

**Table 7:** Moderated chain mediation effect analysis.

Chain mediation path	Level	Effect	SE	95% CI lower	95% CI upper
FWA → KA → IM → TW	Low	0.025	0.009	0.010	0.044
	Mean	0.052	0.014	0.026	0.082
	High	0.080	0.022	0.039	0.126
	Difference	0.056	0.017	0.026	0.092

When CF was at a low level, the indirect effect of FWA on Thriving at Work through Knowledge Acquisition and Intrinsic Motivation was 0.025 [BootSE = 0.009, 95% CI = (0.010, 0.044)]. When CF was at a high level, the chained indirect effect increased to 0.080 [BootSE = 0.022, 95% CI = (0.033, 0.126)]. The difference between the two conditions was 0.056 [BootSE = 0.017, 95% CI = (0.026, 0.092)], and the CI did not include zero, thus indicating a significant difference in the mediation effect across levels of CF.

Moreover, the Index of Moderated Mediation was 0.027 [BootSE = 0.009, 95% CI = (0.011, 0.048)], further confirming the significance of the moderated mediation effect. Therefore, the proposed moderated chain mediation effect was supported, and H7 was confirmed.

## 5 Discussion

This study explored how FWA influences employee thriving through psychological mechanisms. Based on SDT, we developed a chained mediation model linking FWA → Knowledge Acquisition → Intrinsic Motivation → Thriving at Work and incorporated Cognitive Flexibility as a moderating variable. From a systems perspective, the model examined how institutional activation, resource absorption, and motivation formation are connected. The following findings were obtained using three-wave data collected from 427 knowledge workers in China.

First, FWA has a significantly positive effect on employee thriving, thus supporting its role in facilitating ongoing development and a growth-oriented mindset at work.

Second, KA and IM function as mediators in the relationship between FWA and thriving. These two variables form a sequential process, which suggests that thriving tends to occur when employees first absorb institutional resources and then convert them into internal motivation.

Third, Cognitive Flexibility influenced the strength of these relationships. Employees with higher CF benefited more from FWA, ultimately displaying greater levels of KA and IM. These differences were also reflected in the indirect effect of FWA on thriving through the mediating variables.

In summary, the effectiveness of flexible work systems depends not only on their formal design but also on how individuals interpret and respond to them. This study highlights a system-based pathway linking institutional support, learning behavior, motivational states, and thriving outcomes, which can help explain how employees respond differently to the same work conditions. The findings also provide practical guidance for organizations seeking to tailor flexible work policies to accommodate individual cognitive characteristics.

### 5.1 Theoretical contributions

This study addresses the core question of how FWA enhance employee TW. Grounded in SDT, it develops a chained mediation model with KA and IM as core mediators, and incorporates CF as a key moderating variable, thereby advancing the understanding of how flexible systems influence employee outcomes. The main theoretical contributions are as follows:

First, this study extends the outcome framework of FWA from traditional extrinsic indicators to developmental outcomes. Prior research has mainly examined the effects of FWA on job satisfaction, performance, and work–life balance (e.g., McNall et al., 2009; Austin-Egole et al., 2020; Ferdous et al., 2023) while paying limited attention to employees' long-term psychological growth under flexible systems. By adopting TW—characterized by the dual dimensions of vitality and learning—as the outcome variable, this study reveals how FWA promotes employees' self-development and adaptability through knowledge acquisition and motivational activation. These findings broaden the range of outcome variables in FWA research and directly respond to Manuti and Giancaspro's (2019) call for greater attention to employees' psychological growth and internal resources.

Second, this study constructs a chained mechanism model that links institutional activation, psychological need satisfaction, motivational transformation, and outcome generation, with KA and IM serving as central mediating processes. Unlike previous studies that emphasize the direct effects of FWA on employee attitudes (e.g., De Menezes and Kelliher, 2017; Leslie et al., 2012), this study focuses on the psychological dynamics through which institutional resources are absorbed, internalized, and activated. This perspective aligns with Deci and Ryan's (2008) proposition that IM arises from the satisfaction of basic psychological needs. The findings expand the theoretical boundaries of SDT in organizational contexts and provide a mechanism-based explanation of psychological transformation under empowerment-oriented systems.

Finally, this study incorporates CF into the moderating pathway, addressing the theoretical call to consider individual difference conditions in institutional research. In contrast to conventional views that treat institutional resources as universal motivators (e.g., Ko and Kim, 2018), the results show that employees with higher CF are more capable of identifying learning opportunities and building relational networks within flexible systems, thereby facilitating resource absorption and transformation. This study also offers a cognitive-pathway interpretation of the relatedness dimension in SDT and echoes. Martin and Rubin's (1995) argument that CF enhances individuals' capacity to adapt to complex environments. These insights reveal the boundary conditions of individual differences in shaping the effects of FWA.

### 5.2 Practical implications

From a managerial perspective, this study offers several actionable recommendations on how to enhance the effectiveness of FWA and foster employee development.

First, the value of FWA lies not only in providing flexibility but also in whether employees can effectively absorb and actively utilize institutional resources. When implementing FWA, managers should simultaneously develop supportive knowledge and learning environments, such as cross-departmental knowledge-sharing platforms, transparent task collaboration processes, and incentives for self-directed learning. These measures help employees identify, acquire, and apply embedded resources, thereby unlocking their potential for continuous growth.

Second, employee TW is not an automatic outcome of structural empowerment but rather the result of a gradual psychological transformation involving KA and IM. Therefore, the implementation of FWA should focus on employees' motivational pathways by setting meaningful goals, providing timely feedback, establishing coaching mechanisms, and ensuring task autonomy to strengthen their learning intentions and intrinsic growth drive.

Third, CF plays a crucial moderating role in how employees respond to FWA. Organizations should recognize differences in employees' cognitive resources, strategic adjustment capacity, and information processing styles, and adopt tailored support strategies: for employees with lower CF, more structured training and task guidance can be provided; for those with higher CF, greater exploratory space and opportunities for role construction should be offered. Such context—person alignment enhances the fit between institutional design and individual capacity.

Finally, these findings are particularly relevant for knowledge-intensive, remotely coordinated, and high task-uncertainty contexts. In such settings, managers should pay close attention to employees' psychological absorption processes and motivational responses to institutional resources, enabling a win–win outcome for both organizational adaptability and individual development.

### 5.3 Research limitations and recommendations

First, although this study employed a three-wave survey design to mitigate common method bias, all core variables were obtained through self-reports from employees. Such a data source may still be affected by subjective factors such as social desirability bias and memory recall errors, which could reduce the objectivity and accuracy of the findings. Future research could incorporate multi-source data, such as supervisor ratings, peer evaluations, and objective performance indicators, to enable cross-validation and multidimensional testing of variable relationships, thereby enhancing the robustness and reliability of conclusions (Li and Jo, 2025).

Second, the sample of this study comprised knowledge workers from southern China, operating within a Confucian cultural context characterized by high power distance and relational orientation (Chen and Li, 2026). This cultural background may influence how employees perceive and respond to FWA, KA, and TW. In low power distance or individualistic cultural environments, these mechanisms may operate differently. Future research could conduct cross-cultural or multi-country comparative studies to test the external validity of the proposed model and enrich its theoretical interpretation across cultures.

Third, although this study draws upon Self-Determination Theory to explain the compensatory role of cognitive flexibility, relatedness need satisfaction was not directly measured. This omission limits our ability to empirically verify whether cognitive flexibility truly operates by buffering unmet relational needs or through alternative self-regulatory mechanisms. Consequently, the proposed psychological pathway should be interpreted as a theoretically grounded inference rather than a directly validated process. Future research may incorporate explicit measures of relatedness need satisfaction to disentangle these potential mechanisms and provide a more fine-grained understanding of how cognitive resources interact with basic psychological needs in flexible work contexts.

Fourth, this study identified KA and IM as mediating variables and CF as a moderating variable, revealing part of the psychological mechanism linking FWA to TW. However, it did not incorporate a broader range of emotional, cognitive, and contextual factors. Future research could integrate variables such as psychological safety and work meaningfulness into the mediating pathway to better capture the emotional and cognitive bridges between institutional activation and behavioral outcomes. In addition, incorporating individual difference variables (e.g., proactive personality, learning goal orientation) and contextual factors (e.g., task complexity, team structure) as moderators could help build a more integrative and ecologically valid multilevel mechanism model.

## 6 Conclusion

In an era marked by the rapid evolution of organizational structures and employee roles, FWA functions not only as a technical response to enhance organizational adaptability and efficiency, but also as a strategic mechanism for fostering employee potential and supporting continuous individual development. By developing a chained mediation model, this study clarifies how FWA can promote employee thriving through the sequential activation of KA and IM. Furthermore, by introducing CF as a boundary condition, the study extends the understanding of how institutional resources are differentially internalized and transformed across individual cognitive profiles. These findings contribute to the refinement of SDT in the context of modern work systems and provide theoretical grounding for the precision design and differentiated implementation of flexible work policies. Looking ahead, organizations are encouraged to account for the interactions between employees' cognitive traits and psychological mechanisms more deliberately. Aligning system-level design with individual-level adaptability is critical for building organizations that are both resilient and growth-oriented in the face of ongoing change.

## Data Availability

The raw data supporting the conclusions of this article will be made available by the authors, without undue reservation.
